# Fibroblast growth factor-21 alleviates proteasome injury via activation of autophagy flux in Parkinson’s disease

**DOI:** 10.1007/s00221-023-06709-3

**Published:** 2023-11-01

**Authors:** Yufei Shen, Zhuoying Zhu, Yanping Wang, Shuxia Qian, Congying Xu, Baorong Zhang

**Affiliations:** 1 College of Medicine, Zhejiang University, Hangzhou, 310009 China; 2 Department of Neurology, The Second Affiliated Hospital of Jiaxing University, Jiaxing, 314001 China; 3 Department of Neurology, College of Medicine, Second Affiliated Hospital, Zhejiang University, Hangzhou, 310009 China

**Keywords:** Fibroblast growth factor 21, Parkinson’s disease, Neurodegeneration, Autophagy, Proteasome, Ubiquitin–proteasome system

## Abstract

Parkinson’s disease (PD) is one of the most common and complex Neurodegeneration, with an inherited metabolic disorder. Fibroblast growth factor 21 (FGF21), an endocrine hormone that belongs to the fibroblast growth factor superfamily, plays an extensive role in metabolic regulation. However, our understandings of the specific function and mechanisms of FGF21 on PD are still quite limited. Here, we aimed to elucidate the actions and the underlying mechanisms of FGF21 on dopaminergic neurodegeneration using cellular models of parkinsonism. To investigate the effects of FGF21 on dopaminergic neurodegeneration in vitro, proteasome impairment models of PD were utilized. Human dopaminergic neuroblastoma SH-SY5Y cells were treated with the proteasome inhibitor lactacystin (5 μmol/L) for 12 h, then with 50 ng/ml FGF-21 with or without 5 mmol/L of 3-methyladenine.The cells were dissected to assess alterations in autophagy using immunofluorescence, immunoblotting and electron microscopy assays. Our data indicate that FGF21 prevents dopaminergic neuron loss and shows beneficial effects against proteasome impairment induced PD syndrome, indicating it might be a potent candidate for developing novel drugs to deal with PD.

**Supplementary Information:**

The online version contains supplementary material available at 10.1007/s00221-023-06709-3.

## Introduction

Parkinson’s disease (PD) is one of the most common neurodegenerative diseases, which mainly affects the dopaminergic (DA) neuron system in the substantia nigra (SN) of the brain, and causes degeneration over time (Braak et al. 2003). This deterioration will lead to abnormal motor function of patients affected by PD, which will lead to tremor, bradykinesia, dyskinesia, postural instability and slurred speech. Recent studies also suggest a strong association with the development of non-motor dysfunction (Cuervo et al. 2004). The main pathological features of PD are the formation of inclusion bodies in the cell bodies of neurons (LBs) and the aggregation of α-syn folded incorrectly in the process of neurons, which leads to the formation of Lewis neurites (LNs) (Lansbury and Lashuel 2006). α-Syn, a presynaptic protein involved in neurotransmission, is usually degraded by ubiquitin–proteasome system (UPS) and Autophagosome Degradation System (ALS). The main function of UPS is the selective degradation of short-term proteins, while ALS is mainly responsible for the specific proteins elimination (Dawson and Dawson 2003; Lansbury and Lashuel 2006). The dysfunction of UPS can lead to the activation of ALS and enhance the removal of abnormal proteins. The unusually high affinity of mutant α-syn prevented the uptake of lysosomes and inhibited their degradation by ALS (Massey et al. 2006).This shows that ALS dysfunction is an important mechanism of neurodegenerative diseases, especially PD. Subsequently, Blockage of ALS may exacerbate various factors and further complicate PD (Shen et al. 2013).

In recent years, Many studies suggests that metabolic disorders are widely related to neurodegenerative diseases (Chen et al. 2014; Moran et al. 2019; Ristow 2004). Therefore, metabolic regulatory molecules such as insulin (Nelson and Alkon 2005), glucagon-like peptide 1 (Chen et al. 2014, 2019) and fibroblast growth factor 21(FGF21) (Chen et al. 2019) may represent potential pharmacotherapies for neurodegenerative diseases. FGF21 belongs to FGF superfamily, which is composed of 23 members and widely participates in many cellular procession: cell growth, differentiation, wound healing, neuron development and angiogenesis (Beenken and Mohammadi 2009). FGF21 is a secreted protein consisting of 210 amino acids and a hydrophobic amino terminal. FGF21 mainly works through its classic co-receptors β-klotho (β-Klotho) and FGF21 receptor 1 (FGFR1). FGF21 is highly expressed in liver, brain and adipose tissue, and can pass through the blood–brain barrier with high permeability (Luo and McKeehan 2013). FGF21 was first considered as an effective hypoglycemic hormone, which can increase the formation of ketone bodies and the oxidation of fatty acids, thus promoting the increase of glucose and triglyceride levels in obese mice (Cantó and Auwerx 2012). FGFR is expressed at low level in different regions of brain and liver. The role of FGF21 is mainly mediated by the brain, especially in the process of gluconeogenesis. The main function of FGF21 is to increase sympathetic nerve activity, thus increasing energy consumption. In a study, FGF21 transgenic mouse model (β-Klotho floxed/Camk2a-Cre) showed a decrease in energy consumption and an increase in body weight even with standard diet. In addition, mice lacking brain β-Klotho reported a decrease of sympathetic nerve activity in brown adipose tissue (BAT), which could be reversed by intracerebroventricular (ICV) injection of FGF21 (Owen et al. 2014). In another study, the central administration of low-dose FGF21 found that sympathetic nerve activated BAT activation and inguinal fat browning were measured by norepinephrine transformation. These evidences indicate that the existence of FGF21 receptor in brain is related to all central metabolism of FGF21. FGF21 can promote the development of PD pathology through proper autophagy. This may be an attractive field for future research. In addition to FGF21, the other two members who recently showed correlation in PD pathology are FGF2 and FGF20.FGF20 is a neurotrophic factor of DA neurons in rat midbrain. When monkey stem cells differentiated into DA in vitro and transplanted into primate PD model, exogenous FGF20 and FGF2 were used to treat neurons, and the relief of PD-related symptoms was observed (Jiang et al. 2004). FGF20 shows a promising answer in stem cell biology, although it shows some negative results in PD etiology in vivo (Ohmachi et al. 2003). In the classical FGF signal axis, FGFR stimulates FGFR tyrosine kinase. In addition, the activation mode of protein kinase B (AKT) and mitogen-activated protein kinase (MAPK) pathway depends on FGFR substrate 2α (FRS2α) (Kakoty et al. 2020). Autophagy leads to inhibition of differentiation of cardiac progenitor cells. However, it has not been determined whether all other members of FGF superfamily are involved in autophagy regulation in PD pathology. The anti-diabetes and weight loss effects of FGF21 in obesity have been fully proved. Recent animal studies have shown that FGF21 has a strong neuroprotective effect in classic AD and PD models (Takagi et al. 2005; Taliyan et al. 2019). Whether FGF21 has neuroprotective effect due to autophagy of dysfunction deserves further study, which is still an attractive research field.

In this study, we used the UPS injury model of PD to study the effect of FGF21 on dopaminergic neurodegeneration and its potential mechanism in vitro. In this study, the administration of recombinant FGF21 (rFGF21) protein were conducted. We studied the effect of FGF21 on degeneration and autophagy of dopaminergic neurons and its potential mechanism.

## Materials and methods

### Cell culture

SH-SY5Y cells (Cell Bank of Chinese Academy of Sciences, Shanghai, China) were maintained in a humidified incubator with 5% CO_2_ at 37 °C, was supplemented with 10% fetal bovine serum (FBS; HyClone; Thermofisher Scientific, Inc) and 1% penicillin/streptomycin Dulbecco modified Eagle medium (DMEM; Hyclone, Thermofisher Scientific, Inc.). When the cells grow to 70–80% confluence, they were treated with serum-free DMEM for 12 h to synchronize. Protease inhibitor lactacystin (Calbiochem, San Diego, California) was prepared in dimethyl sulfoxide (DMSO), the concentration of the stock solution was 1 mmol, then it was added into the culture to a final concentration of 5μmol/L and treated for 12 h. Drug carrier (0.1% DMSO) was used as treatment control. Then, an experiment was conducted (Shen et al. 2013).

## Drug administration

Proteasome injury SH-SY5Y cells were rinsed three times with PBS and Hanks balanced salt solution were added. Then, the solution was replaced by DMEM with 10% FBS, treated with 50 ng/ml FGF 21(ProteTech Group, Inc., Chicago, IL USA) with or without 5 mmol/L 3-methyladenine (3-MA; MCE, USA), in a humidified incubator at 37 °C, and supplied with 5% CO_2_.

## Viability assays

The viability of SH-SY5Y cells was measured by CCK-8 (Yeasen Biotech Co., Shanghai, China). After treatment, a total of 2 × 10^3^ cells were inoculated into each well of a 96-well plate. According to the manufacturer's plan, 10 μl CCK-8 solution was used to incubate the cells at 37 °C for 4 h, and then the optical density (OD) was measured by microplate reader (*λ* = 450 nm). The average OD of five wells was recorded and the cell viability was calculated. This process is repeated at least three times, The cells in the control group were considered to be 100% viable.

Ad green fluorescent protein (GFP) microtubule-associated proteins 1A/1B light chain 3β (LC3B) autophagy fluorescence labeling adenovirus autophagy assay.

Ad GFP LC3B autophagy fluorescent adenovirus reagent (Beyotime Institute of Biotechnology) with multiplicity of infection of 80 was added to SH-SY5Y cells cultured in 24-well plates (50 μl/well)0.12 h after transfection of 8 × 106 pfu (plaque forming unit) adenovirus. After treatment, the expression of GFP was observed by confocal fluorescence microscope at × 100 magnification. The autophagy flux was evaluated by calculating the number of green spots (Yu et al. 2017), using Image J (version 1.8.0; National Institutes of Health, Bethesda, MD, USA).

## Western blot analysis

Cultured SH-SY5Y cells were rinsed with cold PBS twice and were sonicated in ice-cold RIPA lysis buffer (all from Beyotime Institute of Biotechnology). Cell protein concentrations were measured by BCA method (Beyotime Institute of Biotechnology) according to the manufacturer's plan. A total of 40 µg of protein was loaded on 12% SDS–PAGE gel and then transferred to 0.45 μm polyvinylidene fluoride membrane (Roche Diagnostics Co., Shanghai, China). Following blocking in 5% non-fat milk 1 h at room temperature, the membrane was incubated overnight with primary antibodies (all dilutions were 1:1000) at 4 °C; the following primary antibodies were used: anti-LC3 (microtubule-associated protein 1A/1B light chain 3) (ProteTech Group, Inc.), anti-Beclin-1(ProteTech Group, Inc.), anti-P62(Abcam, Cambridge, UK), anti-phosphatidylinositol 3-kinase (PI3K) catalytic subunit type 3(Vps34; Abcam, Cambridge, UK.), Phospho-mTOR (Ser2448) antibody (CST, Danvers, MA, USA), mTOR antibody (CST) and anti-β-actin (ProteinTech Group, Inc).Then the membrane was incubated with the secondary antibodies at 37 °C for 2 h (HRP conjugated Affinipure goat anti-rabbit IgG;1:10,000; Sigma). Use the SuperSignal Detection Kit (Pierce, USA) to visualize the bands. An automatic chemiluminescence imaging analysis system was used to capture images.

## Electron microscope

Cells were fixed with 2.5% glutaraldehyde in 100 mmol/L PBS, then fixed with 1% OsO_4_, dehydrated, then stained with uranyl acetate and lead citrate, and evaluated by electron microscope (Hatachi TEM system, Japan).

## Statistical analysis

Immunoblots were quantified with ImageJ quantification software. Use GraphPad Prism 5.0 software (graphpad software, Inc., La Jolla, CA, USA) for statistical analysis. All experimental data are expressed as mean standard deviation and *p* < 0.05 was considered as statistically significant. Use one-way analysis of variance (ANOVA) and Bonferroni’s multiple comparison tests to determine statistical significance. Using the Shapiro–Wilk test to check the validity of the distribution assumptions.

## Results

**Fig. 1:**
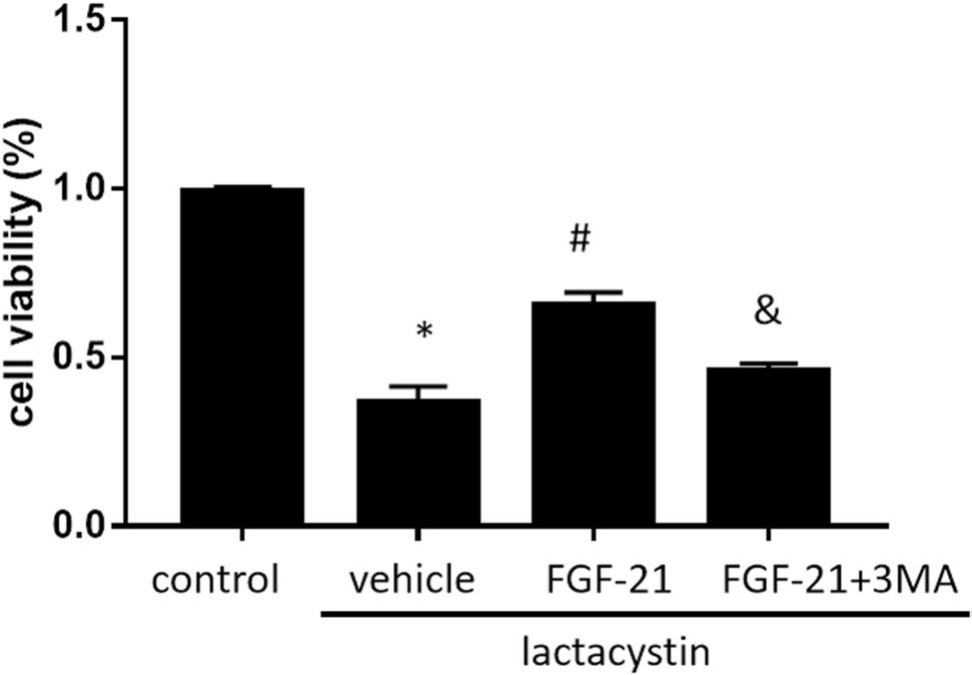
Effects of FGF-21 on cell survival. Effects of FGF-21 (50 ng/ml) on the viability of cultured SH-SY5Y cell during proteasome injury with/without 5 mmol/l 3MA, as determined by the Cell Counting Kit-8 assay.*P<0.05 vs. control group, ^#^*P*<0.05 vs. lactacystin group and ^&^*P*<0.05 vs. lactacystin + FGF-21 group (*n*=5)

Protective effects of FGF21 on proteasome injury. After 12 h of lactacystin treatment, SH-SY5Y cells were cultured in 50ng/ml FGF-21 with/without 5 mmol/l 3MA for 1 h, and CCK-8 assay was performed to measure the cell viability. The results showed that the cell viability of lactacystin group [lactacystin vehicle group = 36.81% ± 4.60%; *p* = 0.000 < 0.05] was significantly lower than that of the control group. FGF-21 treatment attenuated the decrease of cell viability induced by proteasome injury. [Lactacystin + FGF-21(50 ng/ml) group = 65.37% ± 3.88%; *p* = 0.002 < 0.05]. After adding autophagy inhibitor 3-MA, the cell viability was reduced Compared with lactacystin + FGF-21 group [Lactacystin + FGF-21(50 ng/ml) + 5 mmol/l 3MA group = 45.97% ± 5.83%; *p* = 0.000 < 0.05] (Fig. 1). These results indicated that FGF21 significantly alleviates SH-SY5Y proteasome injury induced by lactacystin. 3-MA partially eliminated the effect of FGF-21 on the cell viability of lactacystin + FGF-21 group (Fig. 1). These results indicate that 3-MA may partially reverse the protective effect of FGF-21 on the survival of SH-SY5Y cells during proteasome injury.

**Fig. 2:**
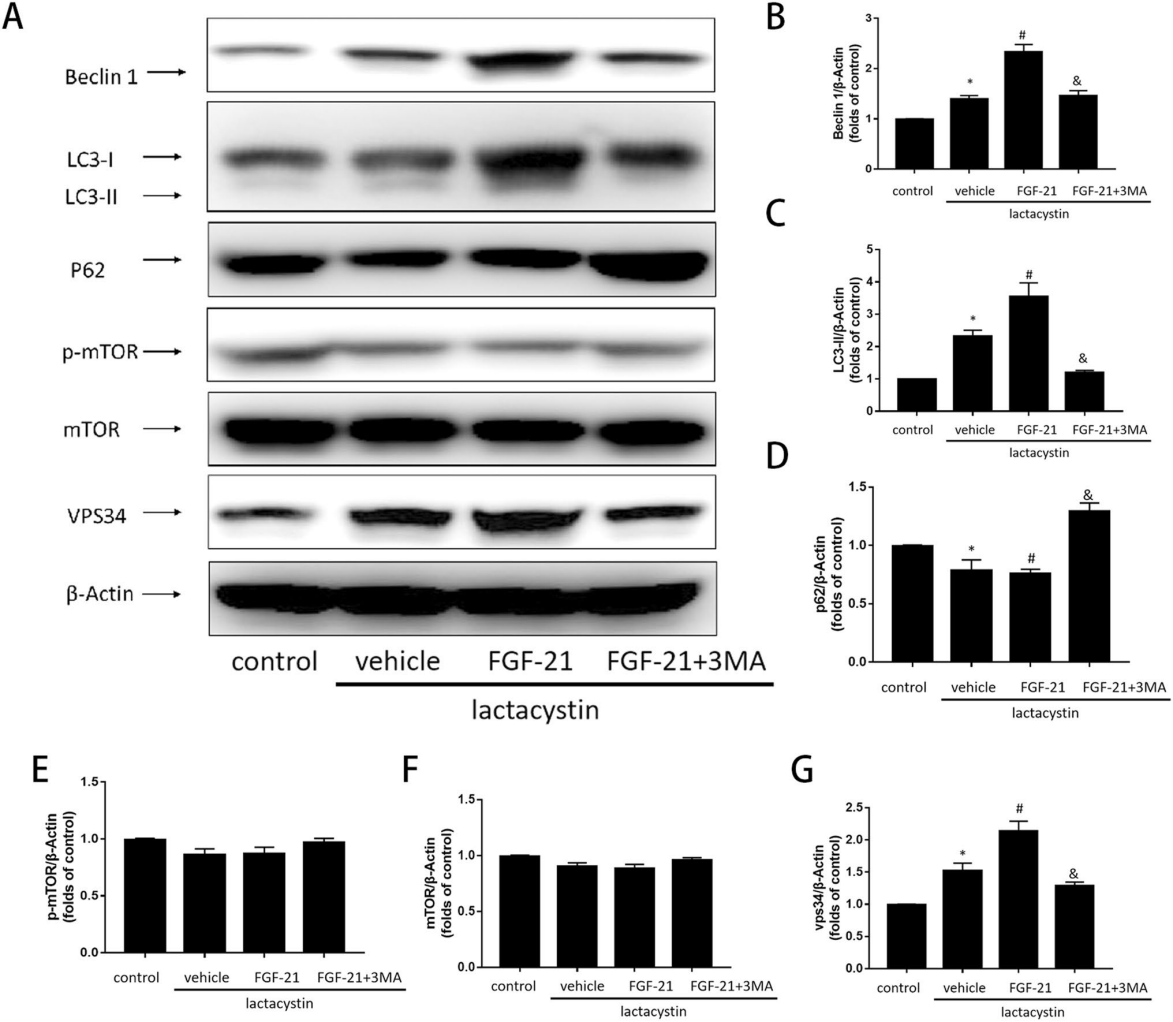
Effects of FGF-21 on autophagy makers in proteasome-damaged SH-SY5Y. **A** Western blot analysis of autophagy makers. **B** Densitometric analysis of Beclin-1 levels. **C** Densitometric analysis of LC3-II levels. **E** Densitometric analysis of p62 levels. **F** Densitometric analysis of mTOR levels. **F** Densitometric analysis of p-mTOR levels. **G** Densitometric analysis of Vps34 levels. *P  <  0.05 vs. control group, ^#^*P*  <  0.05 vs. lactacystin group and ^&^*P*  <  0.05 vs. lactacystin  +  FGF-21 group. Values are presented as mean  ±  standard deviation. Experiments were repeated in triplicate. *FGF 21* fibroblast growth factor 21, *3 MA* 3 methyladenine, *LC3 II* lipid modified microtubule-associated proteins 1A/1B light chain, *p62* + sequestosome 1, *mTOR* mechanistic target of rapamycin, *p* phosphorylated (*n* = 5)

FGF21 enhances autophagy flux of SH-SY5Y cells in proteasome injury model. Western blot analysis indicated that, Compared with the control group, Beclin-1 protein [*p* = 0.001 < 0.05]and the formation of LC3-II [*p* = 0.000 < 0.05] in lactacystin group was significantly increased. Compared with the control group, the expression of p62 [*p* = 0.017 < 0.05] decreased significantly. P62 is a kind of receptor, which can maintain cell homeostasis by interacting with autophagy cargo and LC3 protein at the same time, thus promoting selective autophagy (Pankiv et al. 2007). Compared with lactacystin group, in lactacystin + FGF-21 group, the level of Beclin-1 protein [*p* = 0.000 < 0.05] and the formation of LC3-II increased [*p* = 0.011 < 0.05], and P62 [*p* = 0.036 < 0.05]expression decreased correspondingly. However, compared with lactacystin + FGF-21 group, after 3-MA treatment, the expression of Beclin-1 protein [*p* = 0.001 < 0.05] and the formation of LC3-II [*p* = 0.000 < 0.05] were down-regulated and the expression of p62 [*p* = 0.000 < 0.05] was up-regulated (Fig. 2B, C, D), which indicated that 3-MA partially inhibited FGF-21-induced autophagy.

The effect of FGF21 is related to the change of mTOR and Vps34 signals induced by lactacystin. Acetylcholine can activate autophagy through AMPK–mTOR pathway and increase the tolerance of cells to proteasome injury. A preliminary study showed that, FGF21 plays a neuroprotective role in ApoE-KO mice with long-term calorie restriction by prolonging the activation of AMPK–mTOR signaling pathway (Rühlmann et al. 2016). Then, no significant difference was observed among the control group, lactacystin and lactacystin + FGF-21 group (Fig. 2E, F), indicating that FGF-21-induced autophagy may not occur via mTOR signaling pathway. The Beclin-1/Vps34 complex formed by Vps34 and Beclin-1 can regulate the formation of autophagy membrane (Yang et al. 2019). This study demonstrated that FGF-21 increased the expression level of Beclin-1 protein in proteasome injury cell (Fig. 2B). The autophagy inhibitor 3-MA used in this study is the inhibitor of Vps34. Therefore, the expression levels of Vps34 protein were examined in each group. The results showed that the expression level of Vsp34 protein [*p* = 0.001 < 0.05] in lactacystin group was significantly higher than that in control group. While lactacystin + FGF-21 group [*p* = 0.000 < 0.05]increased further. However, compared with lactacystin + FGF-21 group, the co-treatment of FGF-21 and 3-MA reduced the expression level of Vsp34 protein [*p* = 0.005 < 0.05]. In addition, compared with lactacystin group, the expression level of Vsp34 protein [*p* = 0.001 < 0.05] in lactacystin + 3-MA group was significantly reduced (Fig. 2G). These results indicate that Vps34 protein plays a role in FGF-21 enhancing autophagy of proteasome-damaged cells.

**Fig. 3:**
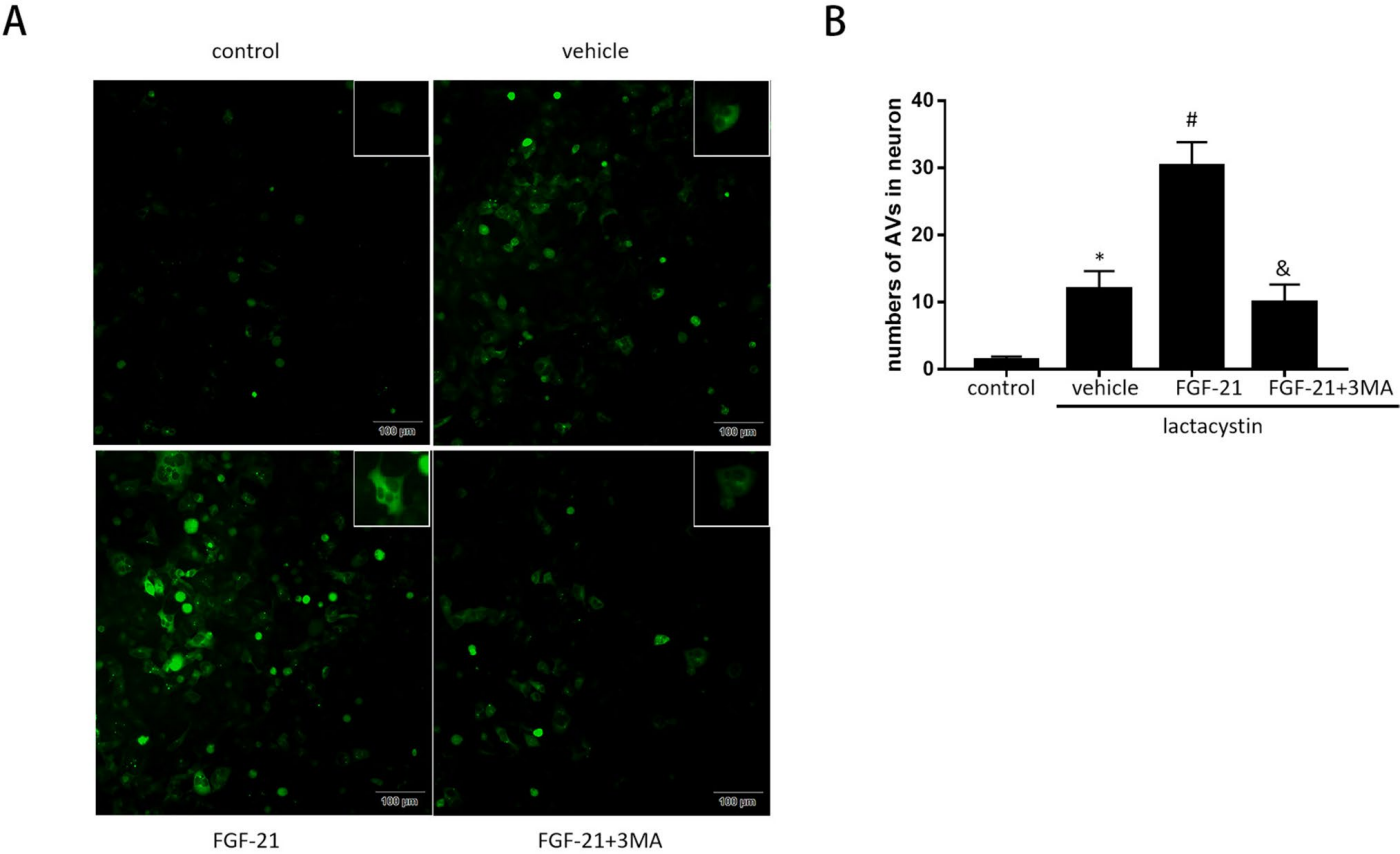
Effects of FGF-21 on autophagic flux in proteasome-damaged SH-SY5Y. **A** SH-SY5Y transfected with adenovirus harboring tandem fluorescent GFP-LC3 (Ad-LC3-SH-SY5Y) for 24 h were subjected to different treatments. Representative images of immunofluorescent SH-SY5Y expressing GFP-LC3. Green fluorescence represents GFP. **B** Semi-quantitative analysis of autophagosomes (Green dots). *P  <  0.05 vs. control group, ^#^*P*  <  0.05 vs. lactacystin group and ^&^*P*  <  0.05 vs. lactacystin  +  FGF-21 group. Values are expressed as mean ± standard. Experiments were repeated in triplicate. *FGF-21* fibroblast growth factor-21, *3-MA* 3-methyladenine, *GFP* green fluorescent protein (*n*  =  5)

To monitor autophagy flux, serial fluorescence GFP-LC3B was performed on SH-SY5Y cells (Ad-LC3-SH-SY5Y). In the control group, Ad-LC3-SH-SY5Y cells showed basic autophagy with few autophagies and lysosomes. The Ad-LC3-SH-SY5Y cells in lactacystin treatment group showed increased autophagy and less autophagy [*p* = 0.002 < 0.05], indicating that autophagy flux increased during proteasome injury. In lactacystin + FGF-21 group, compared with lactacystin group, Ad-LC3-SH-SY5Y cells receiving FGF-21 had more autophagy and lysosomes [*p* = 0.001 < 0.05] indicated that FGF-21 treatment additionally enhanced autophagy flux. However, compared with FGF-21 treatment group, the combined treatment of FGF-21 and autophagy inhibitor 3-MA reduced autophagy and lysosome [*p* = 0.001 < 0.05], indicating that FGF-21 treatment enhanced autophagy flux and could be partially inhibited by 3-MA (Fig. 3). These data indicate that FGF21 induces up-regulation of autophagy flux during proteasome injury. FGF21-mediated autophagy enhances cell survival.

**Fig. 4:**
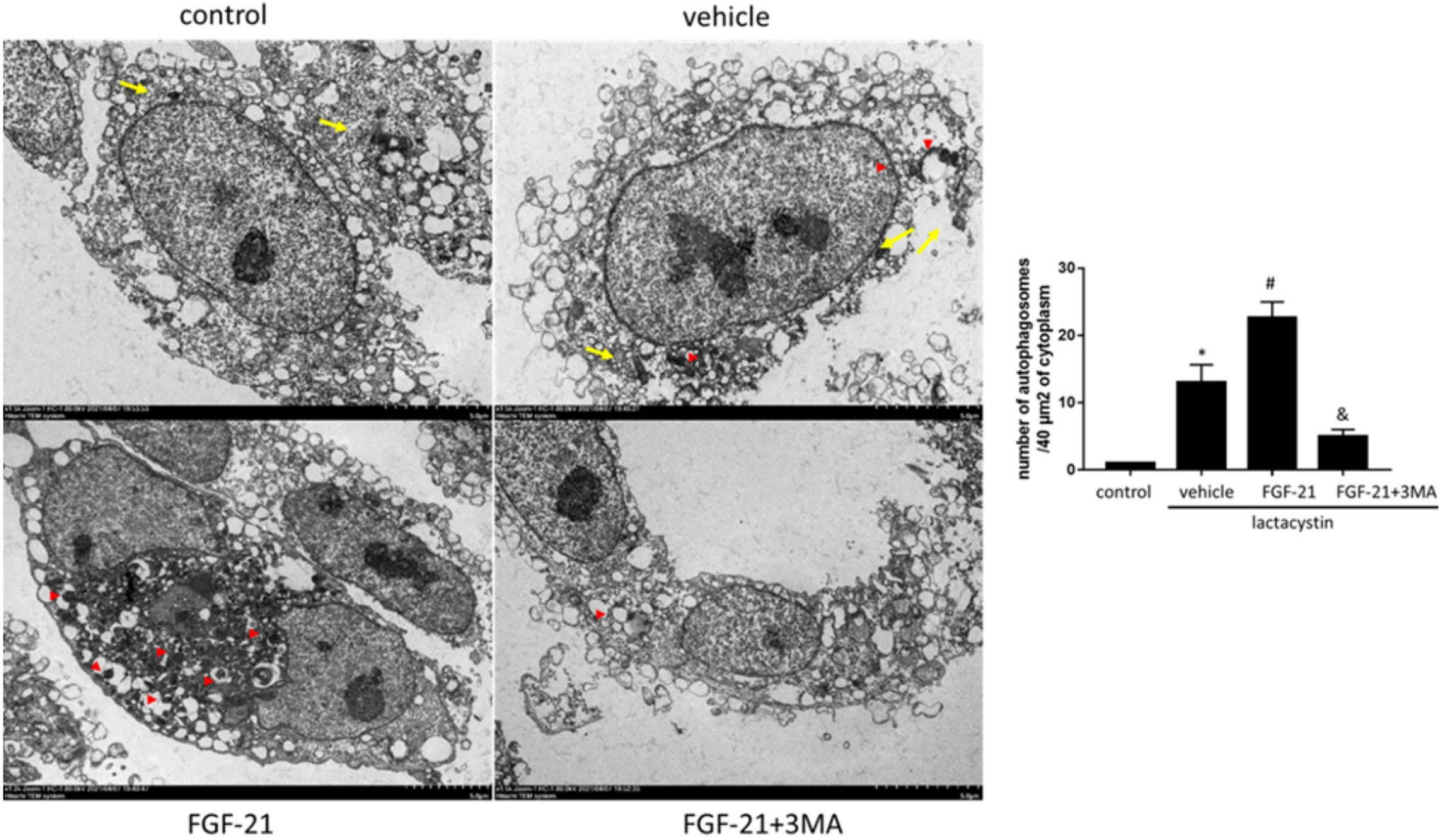
Changes of organelles and autophagosomes in proteasome-damaged SH-SY5Yassessed by transmission electron microscopy (yellow arrow: mitochondria, red triangle: autophagosomes). Analysis chart: the number of autophagosomes was counted every 40 µm^2^ (10 fields). *P  <  0.05 vs. control group, ^#^*P*  <  0.05 vs. lactacystin group and ^&^*P*  <  0.05 vs. lactacystin + FGF-21 group. Values are presented as mean ± standard deviation (*n*  = 3)

In the electron microscope images, we also observed alteration with autophagy, compared with lactacystin group, in lactacystin + FGF-21 group, more autophagy characterized by double membrane structure devoured intracellular organelles [*p* = 0.008 < 0.05], indicating that FGF-21 administration induced enhanced autophagy formation and Autophagosomes (Fig. 4).

## Discussion

UPS impairment models are designed to evaluate the role of autophagy in the etiopathogenesis of PD. At present, many studies have used this cell model to study the mechanism of autophagy in the pathogenesis of PD (Shen et al. 2013; Xie et al. 2010; Zhang et al. 2005). In this study, compared with the control group, the survival rate of neurons in lactacystin group decreased significantly, indicating that UPS injury model was successfully established. Many studies have demonstrated that autophagy level in PD model increases. At present, it is still unclear whether autophagy has protective or deleterious role in PD neuropathology. Low levels of autophagy induced by moderate hypoxia or ischemia have protective effects and may prevent activation of apoptosis by degrading and removing damaged Mitochondria (Decker et al. 1980). It has also been suggested that autophagy may be a compensation mechanism and play a protective role by degrading potentially toxic protein.

In this study, we determined whether the protective effect of FGF21 on dopaminergic neurons is related to autophagy. We found that the number of lysosomes increased, the autophagy flux increased and the mitochondrial damage decreased significantly in FGF21-treated cells, which indicated that FGF21 might mediate lysosome-induced autophagy, reduce mitochondrial damage and reduce the incidence of apoptosis.

Studies have shown that autophagy may be regulated by several signal pathways, such as Beclin-11Vps34 complex (Yang et al. 2019), AMPKK–mTOR and PI3KK–AKTT–mTOR (Ling et al. 2016; Zhao et al. 2013). Studies have proved that FGF-21 may play a neuroprotective role by activating AMPKK–mTOR signal transduction pathway in ApoE-KO mice with long-term heat limitation (Rühlmann et al. 2016). However, in the experimental results of this study, p-mTOR and mTOR protein did not change significantly after treated with FGF-21 after proteasome injury. It shows that FGF-21 may enhance autophagy through other pathways than mTOR pathway and protect nerve cells from damage. Beclin-1 is the earliest mammalian autophagy gene located on human chromosome 17q21(40) (Aita et al. 1999). The expression of Beclin-1 in Golgi apparatus mainly regulates autophagy-related proteins by forming Vps34 complex (Yang et al. 2019), and locates these autophagy-related proteins in the precursor structure. In this study, when 3-MA was used to inhibit the expression of autophagy-related genes Beclin-1 and Vps34, compared with lactacystin + FGF-21 treatment group, the nerve cell damage in lactacystin + FGF-21 + 3-MA group was significantly increased, and similar results were observed in the expression of autophagy-related proteins, which indicated that FGF-21-related neuroprotection was enhanced by Beclin–11Vps34 complex. However, the crosstalk mechanism between autophagy and Vps34 is still unclear, which should be discussed in future research.

The purpose of this study is to study the protective effect of exogenous FGF21 on dopaminergic neurons and explore its possible mechanism. There are still some shortcomings in this study: because there is no specific agonist or inhibitor of FGF21 reported at present, the expression levels of FGF21 protein and mRNA in UPS impairment model cells have not been detected. In addition, there is no in vivo experiments to improve the understanding of the role of FGF21 in PD at present, Whether FGF21 can exhibit neuroprotective effect in vivo is worth further study and remains an attractive area of research, with the hope that it could specifically block PD progression.

### Supplementary Information

Below is the link to the electronic supplementary material.

Supplementary file1 (JPG 66 KB)

Supplementary file2 (JPG 56 KB)

Supplementary file3 (JPG 30 KB)

Supplementary file4 (JPG 19 KB)

Supplementary file5 (JPG 46 KB)

Supplementary file6 (JPG 60 KB)

Supplementary file7 (JPG 24 KB)

## Data Availability

All data generated or analyzed during this study are included in this published article.

## Abbreviation

PD: Parkinson’s disease
FGF21: Fibroblast growth factor 21
DA: Dopaminergic
SN: Substantia nigra
LNs: Lewis neurites
UPS: Ubiquitin–proteasome system
ALS: Autophagosome Degradation System
FGFR1: FGF21 receptor 1
BAT: Brown adipose tissue
ICV: Intracerebroventricular
AKT: Protein kinase B
MAPK: Mitogen-activated protein kinase
FRS2α: FGFR substrate 2α
rFGF21: Recombinant FGF21
FBS: Fetal bovine serum
DMEM: Dulbecco modified Eagle medium
DMSO: Dimethyl sulfoxide
OD: Optical density
LC3B: Light chain 3β
ANOVA: Analysis of variance
