# Supplementary figures and images for: Fibroblast growth factor-21 alleviates proteasome injury via activation of autophagy flux in Parkinson’s disease
Source: Exp Brain Res. 2023 Nov 1;242(1):25–32. doi: 10.1007/s00221-023-06709-3 (PMC10786996; doi:10.1007/s00221-023-06709-3)

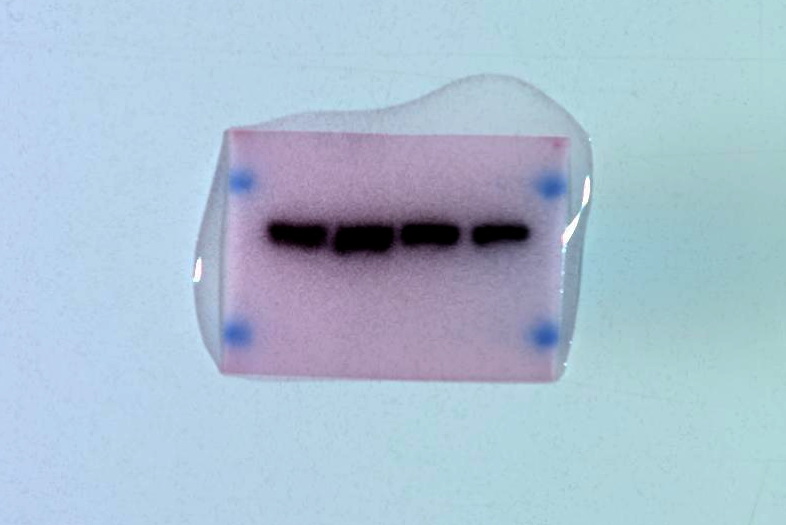

Supplement: Supplementary file 1 — Supplementary file1 (JPG 66 KB) [file 221_2023_6709_MOESM1_ESM.jpg]

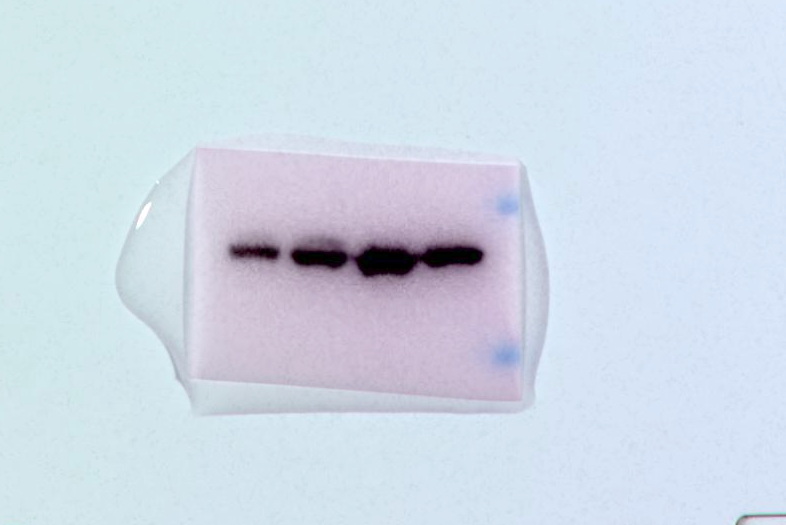

Supplement: Supplementary file 2 — Supplementary file2 (JPG 56 KB) [file 221_2023_6709_MOESM2_ESM.jpg]

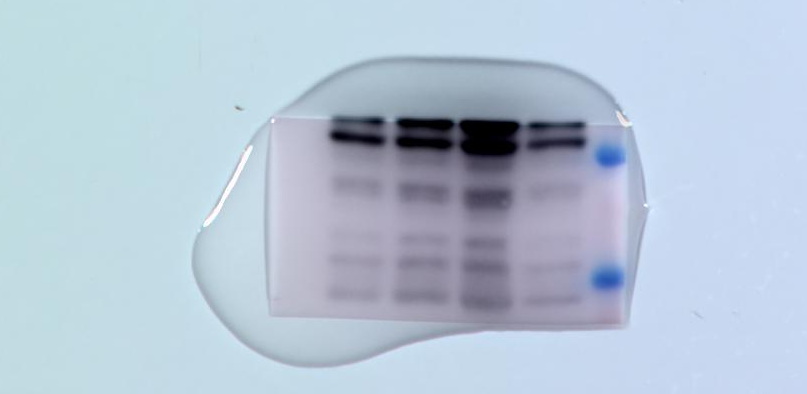

Supplement: Supplementary file 3 — Supplementary file3 (JPG 30 KB) [file 221_2023_6709_MOESM3_ESM.jpg]

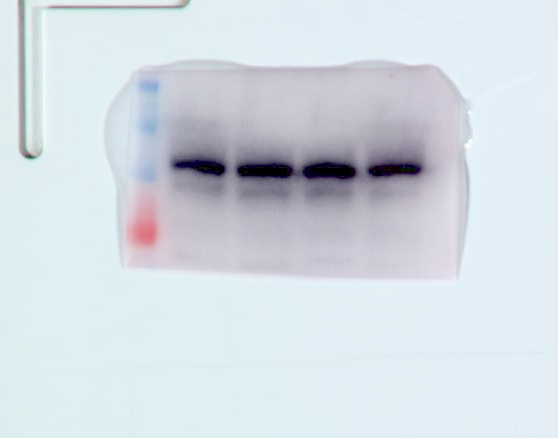

Supplement: Supplementary file 4 — Supplementary file4 (JPG 19 KB) [file 221_2023_6709_MOESM4_ESM.jpg]

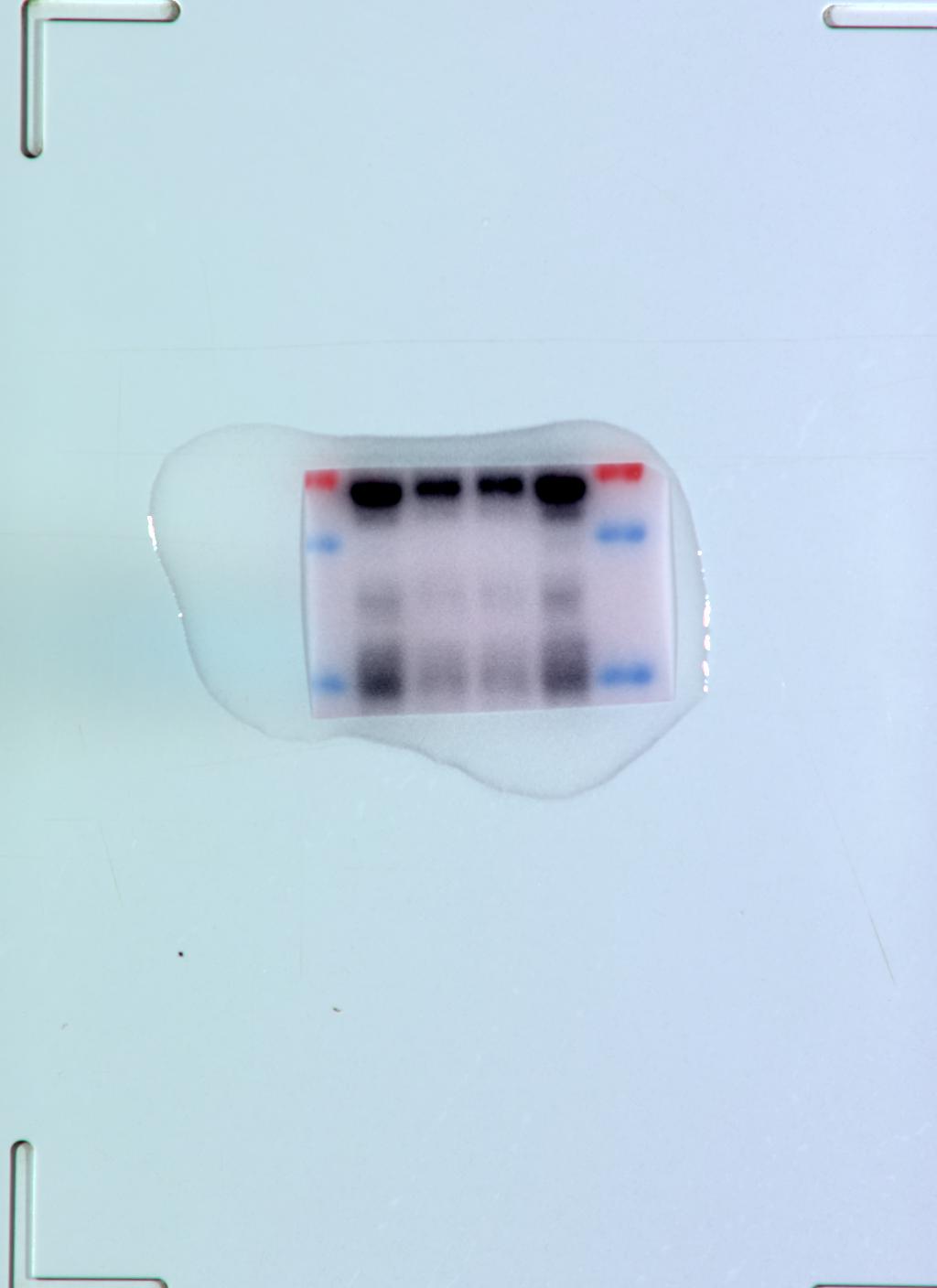

Supplement: Supplementary file 5 — Supplementary file5 (JPG 46 KB) [file 221_2023_6709_MOESM5_ESM.jpg]

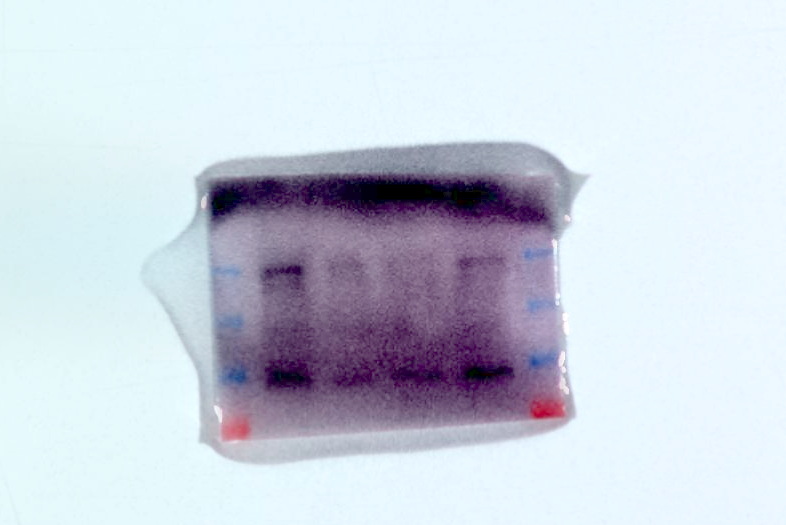

Supplement: Supplementary file 6 — Supplementary file6 (JPG 60 KB) [file 221_2023_6709_MOESM6_ESM.jpg]

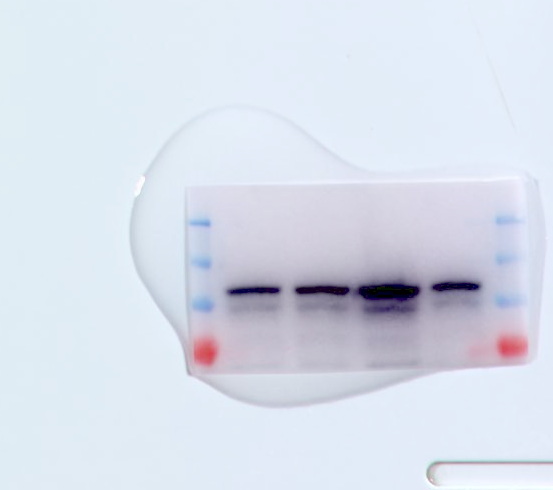

Supplement: Supplementary file 7 — Supplementary file7 (JPG 24 KB) [file 221_2023_6709_MOESM7_ESM.jpg]
